# Revision of Tibiotalar Arthrodesis Nonunion Using Intramedullary Fibular Autograft Combined with Cancellous Iliac Graft and Bone Marrow Aspirate Concentrate: A Case Report and Literature Narrative Review

**DOI:** 10.3390/jcm15052078

**Published:** 2026-03-09

**Authors:** Daniele Marcolli, Alice Montagna, Elena Delmastro, Antonio Mazzotti, Carlo Francesco Minoli, Paolo Ferrua, Pietro Simone Randelli

**Affiliations:** 1 Università degli Studi di Milano, 20122 Milan, Italy; daniele.marcolli@asst-pini-cto.it; 2 Unità Operativa Semplice Dipartimentale Centro Patologia del Piede (CPP), ASST Gaetano Pini-CTO, 20122 Milan, Italy; elena.delmastro@gmail.com; 3 Clinica Ortopedica e Traumatologica Fondazione IRCCS Policlinico San Matteo, 27100 Pavia, Italy; 4 1st Orthopaedics and Traumatologic Clinic, IRCCS Istituto Ortopedico Rizzoli, 40136 Bologna, Italy; antonio.mazzotti4@unibo.it; 5 Unità Operativca Complessa Week Surgery, ASST G. Pini-CTO, 20122 Milan, Italy; carlofrancesco.minoli@asst-pini-cto.it; 6 1^ Clinica Ortopedica, ASST Centro Specialistico Ortopedico Traumatologico Gaetano Pini-CTO, 20122 Milan, Italy; paolo.ferrua@asst-pini-cto.it (P.F.); pietro.randelli@unimi.it (P.S.R.); 7 Laboratory of Applied Biomechanics, Department of Biomedical Sciences for Health, Università degli Studi di Milano, 20122 Milan, Italy; 8 Department of Biomedical, Surgical and Dental Sciences, Università degli Studi di Milano, 20122 Milan, Italy; 9 Research Center for Adult and Pediatric Rheumatic Diseases (RECAP-RD), Department of Biomedical Sciences for Health, Università degli Studi di Milano, 20122 Milan, Italy

**Keywords:** ankle arthrodesis, revision surgery, nonunion, intramedullary fibular autograft, bone marrow aspirate concentrate, computed tomography

## Abstract

**Background/Objectives**: Nonunion after tibiotalocalcaneal (TTC) arthrodesis remains challenging, especially in revision settings where union rates are substantially lower than in primary procedures. Biological adjuncts are commonly used to enhance healing, yet most described methods employ fibular onlay struts and cancellous autograft. To our knowledge, intramedullary placement of a fibular autograft for ankle fusion has not previously been reported. This study presents a revision of TTC arthrodesis nonunion treated with this technique and summarizes existing evidence on revision ankle arthrodesis, fibular grafting, and bone marrow aspirate concentrate (BMAC). **Methods**: We report a revision TTC arthrodesis nonunion managed with a decorticated intramedullary fibular autograft spanning the tibiotalar canal, supplemented with cancellous iliac crest autograft and BMAC. A review of PubMed, Scopus, and Google Scholar (search date: 1 September 2025) was performed to identify studies addressing revision ankle fusion, fibular grafting techniques, and BMAC use in foot and ankle arthrodesis. Primary outcomes included union and complications, with CT-based assessment prioritized when available. **Results**: At 3 months, radiographs and CT demonstrated progressive osseous bridging consistent with fusion; the patient achieved pain-free weight-bearing without complications. **Conclusions**: Intramedullary fibular autograft in revision TTC arthrodesis is a novel biological-mechanical strategy that leverages endosteal contact and axial stability while augmenting osteogenesis with cancellous autograft and BMAC. The review supports the biological plausibility and safety of this approach and underscores the importance of CT-based assessment.

## 1. Introduction

Tibiotalocalcaneal (TTC) arthrodesis is a widely accepted salvage procedure for end-stage ankle arthritis, severe deformity, avascular necrosis of the talus, failed total ankle arthroplasty, Charcot neuroarthropathy, and complex post-traumatic conditions [1]. By achieving fusion of the tibiotalar and subtalar joints, TTC arthrodesis aims to restore a stable, plantigrade, and pain-free limb, allowing for functional ambulation in patients with otherwise disabling pathology. Over the past decades, advances in fixation techniques—including retrograde intramedullary nails, locking plates, and compression screw constructs—together with improved perioperative protocols, have enhanced union rates and reduced complication profiles [2]. Nevertheless, nonunion remains a clinically relevant and challenging complication, with reported rates varying widely depending on patient comorbidities, bone quality, surgical technique, and definition criteria [3,4,5]. Primary TTC arthrodesis typically achieves fusion rates approximating 90–95%, whereas revision procedures face higher biological and mechanical hurdles, with reported union rates commonly in the 70–85% range and complication rates that may exceed those of primary fusion [6,7]. Persistent nonunion compromises pain relief, gait and patient-reported outcomes [8,9,10].

Contemporary strategies to improve fusion emphasize both mechanical stability and biological enhancement [11,12]. Autologous iliac crest cancellous bone graft (ICBG) remains the reference standard, yet donor-site morbidity and volume constraints motivate adjuncts. Bone marrow aspirate concentrate (BMAC) has emerged as a safe source of osteogenic cells and signaling molecules and is increasingly applied in foot and ankle fusions [13,14,15]. Fibular grafts have a long history in ankle arthrodesis, most commonly as onlay struts or “biological plates” in transfibular approaches; however, a proportional meta-analysis suggests that routine addition of fibular onlay to screw constructs may not significantly reduce nonunion in primary TTC arthrodesis [16,17,18]. The role of fibular augmentation may therefore be selective—particularly in revisions with compromised biology or bone stock [19,20]. While meta-analyses suggest no significant benefit of fibular onlay grafts in primary TTC arthrodesis, an intramedullary fibular graft may offer distinct advantages in revision or complex scenarios. By functioning as a structural strut spanning the fusion site, it provides immediate axial and rotational stability, maintains alignment, and acts as a biological scaffold promoting osteointegration, which may be particularly valuable when bone stock is compromised or nonunion risk is high.

In long-bone nonunions, the ipsilateral fibula has been used as a structural autograft—functioning as a “biological intramedullary nail”—to provide axial support and extensive endosteal osteoconduction [20]. Extrapolating this concept to ankle fusion is appealing: an intramedullary fibular autograft can span the tibiotalar canal, increase contact surface, and share load while cancellous autograft and BMAC deliver osteogenic and osteoinductive cues. To our knowledge, such an application has not been reported in the ankle. We therefore present a revision TTC arthrodesis nonunion treated with this combination and contextualize the approach with a literature review focused on revision ankle fusion, fibular grafting and BMAC.

## 2. Case Report

**Figure 1:**
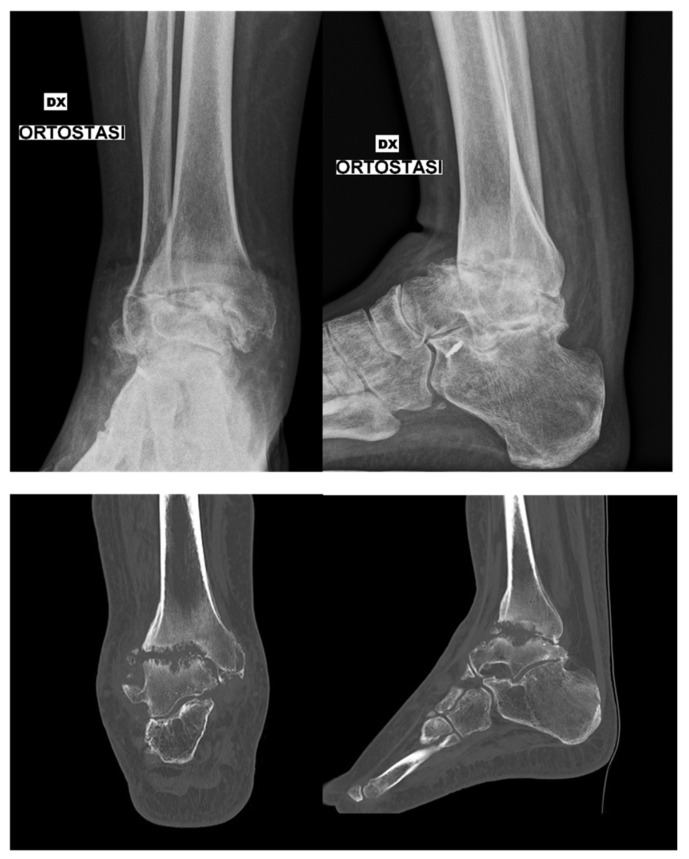
Preoperative imaging: weight-bearing anteroposterior (AP) and lateral radiographs of the ankle and hindfoot, along with corresponding computed tomography (CT) scans in AP and lateral planes, demonstrating post-traumatic arthritic changes.

**Figure 2:**
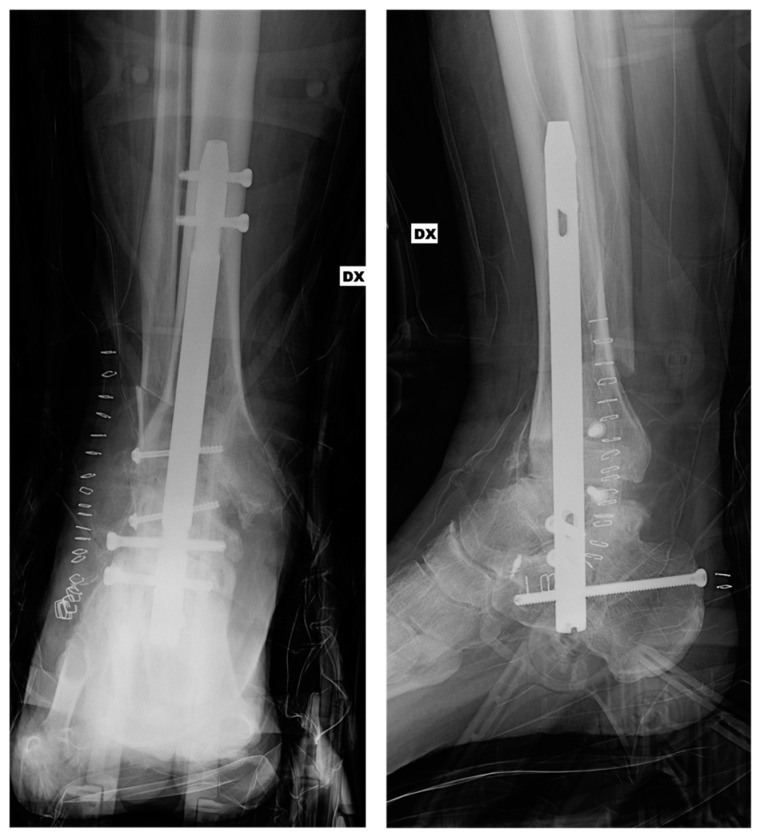
Immediate postoperative anteroposterior (AP) and lateral radiographs.

**Figure 3:**
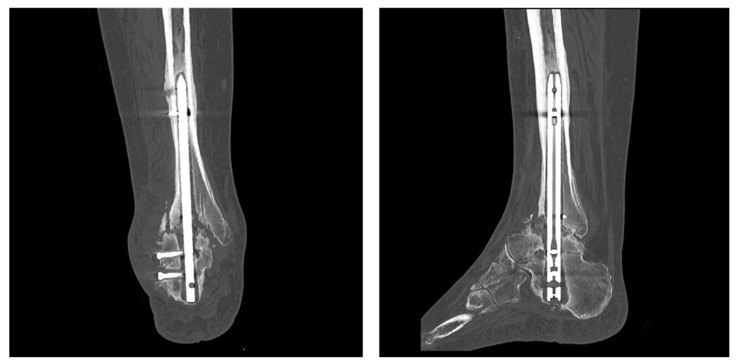
Anteroposterior (AP) and lateral computed tomography (CT) scans obtained 10 months after surgery, demonstrating limited osseous bridging with incomplete consolidation at the tibiotalar and subtalar fusion sites.

The patient was a 45-year-old male, non-smoker, who underwent primary TTC arthrodesis with a retrograde nail for post-traumatic arthritis (Figure 1 and Figure 2). The patient had no relevant comorbidities (including diabetes mellitus). Despite appropriate immobilization and protected weight-bearing, he reported persistent pain—exacerbated by torsional stress—and swelling around the ankle. At 6 months postoperatively, radiographs revealed a persistent lucency across the fusion site and hardware integrity; computed tomography (CT) confirmed lack of bridging bone consistent with pseudoarthrosis (Figure 3).

To address hardware-related symptoms, the patient underwent two minor revision procedures: on 31 January 2025, removal of a distal interlocking screw was performed through the previous surgical approach, followed by accurate hemostasis and layered closure. On 8 July 2025, the remaining proximal screw was removed under fluoroscopic guidance through the same incision, again followed by irrigation and closure. Persistent symptoms and radiological evidence of nonunion prompted a definitive revision procedure.

Infection was carefully excluded pre- and intraoperatively through normal inflammatory markers (CRP, ESR) and negative culture sampling, and no clinical or histological signs of infection were identified.

**Figure 4:**
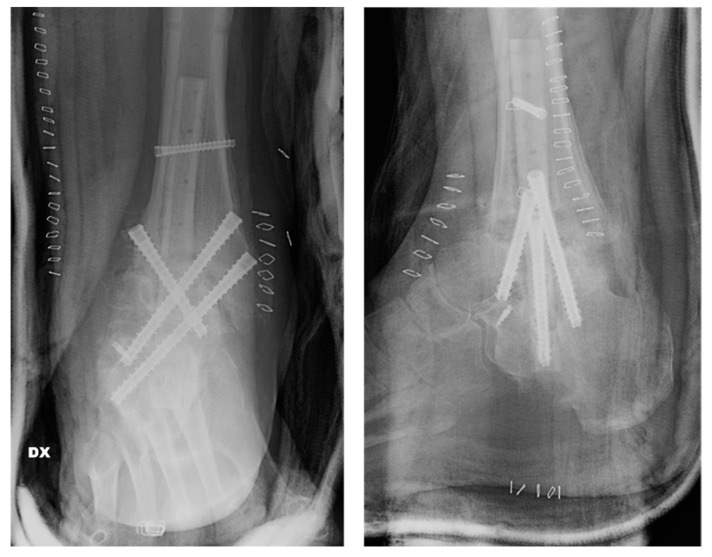
Immediate postoperative anteroposterior (AP) and lateral radiographs following revision TTC re-arthrodesis, demonstrating correct alignment, intramedullary fibular graft positioning, and stable screw fixation.

On 14 August 2025, the procedure was performed through the patient’s previous lateral and medial incisions. The retrograde intramedullary nail and any residual interlocking screws were carefully removed. The nonunion site was meticulously debrided to bleeding bone while preserving viable host surfaces. The intramedullary canals of the distal tibia and talus were prepared, with reaming performed as needed to match the dimensions of the fibular graft and ensure a snug fit. An ipsilateral fibular segment was harvested through the same lateral approach, decorticated, and contoured to fit the tibiotalar canal, with the graft length tailored to span the fusion site. The fibular strut was then introduced intramedullary across the fusion site, functioning as a biological fibular nail to provide axial and rotational stability. Cancellous autograft was harvested from the iliac crest, and bone marrow aspirate concentrate (BMAC) was prepared. Both the fibular graft and cancellous bone chips were soaked in BMAC and implanted circumferentially around the graft at the tibiotalar and subtalar fusion sites to enhance osteointegration. Definitive fixation was achieved using three 7 mm cannulated compression screws (Acutrak^®^) placed from the tibia into the talus, passing through and stabilizing the fibular autograft (Figure 4).

**Figure 5:**
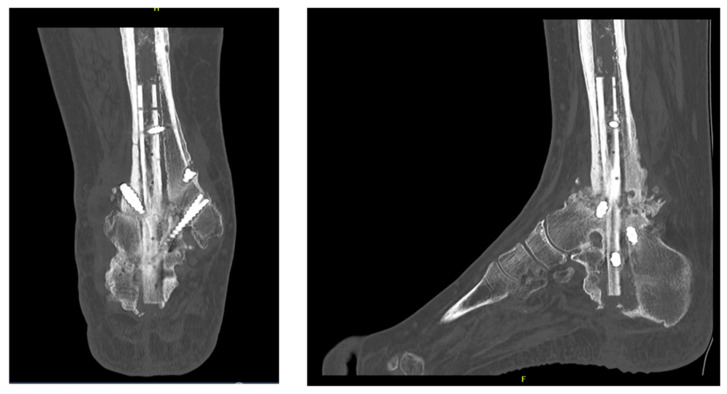
Computed tomography (CT) scan obtained 3 months after revision TTC re-arthrodesis, shown in coronal and sagittal views.

Postoperative protocol: The patient was immobilized in a walker boot and maintained non-weight-bearing for six weeks. A staged progression to partial and then full weight-bearing in axial alignment was undertaken once early radiographic consolidation was observed. Adjunct pulsed electromagnetic field therapy was used overnight according to institutional practice. At 3 follow-up months, CT demonstrated progressive osseous bridging; clinically, the patient reported pain-free weight-bearing and return to daily activities without complications (Figure 5).

## 3. Literature Review

### 3.1. Methods

Search strategy and selection: A systematic search of PubMed, Scopus and Google Scholar was conducted on 1 September 2025 using combinations of terms related to ankle arthrodesis and nonunion (“revision ankle arthrodesis”, “tibiotalar arthrodesis nonunion”, “tibiotalocalcaneal fusion”), fibular grafting (“fibular onlay”, “transfibular”, “fibular graft”), and biological adjuncts (“bone marrow aspirate concentrate”, “BMAC”). Titles and abstracts were screened, followed by full-text review according to the following inclusion criteria: (1) adult ankle/retrohindfoot fusion (primary or revision), (2) outcomes reporting union and/or complications, and (3) description of fibular grafting and/or use of BMAC. Non-English studies, studies without relevant outcomes, and duplicates were excluded. When available, CT-based definitions of union were preferred over radiographic criteria due to known discrepancies (Table 1).

**Figure 6:**
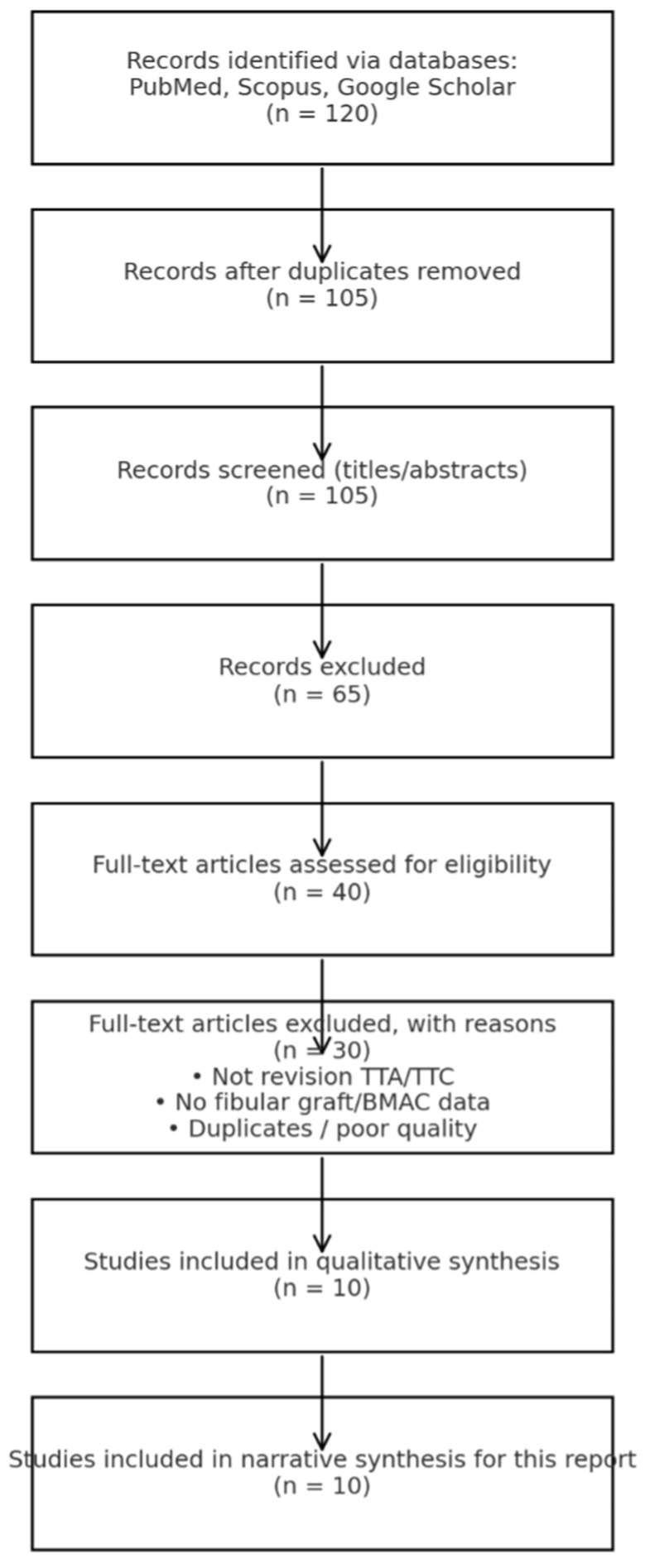
PRISMA flow diagram.

Outcomes and synthesis: The primary outcome was union rate; secondary outcomes included complications, time to union and donor-site morbidity. Given anticipated heterogeneity in patient populations, fixation constructs and definitions of fusion, a narrative synthesis was performed. A PRISMA-style flow diagram summarizes the screening process using simulated counts appropriate for a case-linked mini-review (Figure 6).

### 3.2. Results and Synthesis

Union rates in primary vs. revision ankle fusion: Umbrella and systematic reviews place overall nonunion in foot and ankle arthrodeses near 8%, with single-joint fusions around 6%. However, CT-verified union rates may be lower than plain-film estimates, underscoring the need for standardized imaging. Revision TTA series typically report union in the 80–89% range, though rates vary with fixation strategy, host factors and biological augmentation.

Fixation constructs: Internal fixation using screws or plates remains common in TTC arthrodesis; retrograde nails are frequently used for TTC constructs and for salvage of failed fusions. External ring fixation serves as a valuable option in the presence of infection, bone loss or deformity, with acceptable union rates but higher pin-site complication profiles. Across constructs, smoking and poor local biology consistently predict nonunion.

Fibular grafts—onlay and transfibular techniques: Most ankle fusion literature describes the fibula as a lateral onlay strut or as part of a transfibular approach that creates a “biological plate”. A proportional meta-analysis found that adding fibular onlay to cannulated screw constructs did not significantly reduce nonunion in primary TTC arthrodesis compared with cancellous graft alone. Nevertheless, several retrospective series report high union and favorable alignment with transfibular biological plates, particularly in complex deformity. The overall quality of evidence remains moderate to low, with heterogeneity and limited comparative data.

Intramedullary fibular autograft—evidence base and rationale: In contrast to ankle literature, intramedullary use of the fibula is well described in long-bone nonunions (tibia), where a free fibular strut functions as a “biological nail” delivering axial stability and extensive endosteal osteoconduction; union rates exceeding 85–90% have been reported in selected cohorts. Translating this principle to TTC arthrodesis revision is biologically logical: an intramedullary fibular autograft can bridge the tibiotalar canal, resist shear, enlarge the graft-host interface and provide a scaffold for creeping substitution. To our knowledge, no prior reports have detailed its use in ankle arthrodesis—constituting the novelty of the present case.

BMAC as a biological adjunct in foot/ankle fusion: Topical reviews and clinical series indicate that BMAC is safe and may enhance union in foot/ankle fusions, with reported rates often 75–90% in high-risk cohorts. In diabetic ankle fusions, a historical comparison suggested higher union with BMAC versus iliac crest bone graft alone. Donor-site morbidity after iliac crest harvest for BMAC is generally low in contemporary series; calcaneal harvest has also been described with acceptable profiles. Although randomized comparative data remain scarce, the cumulative evidence supports the plausibility and safety of BMAC as part of a multimodal strategy in revisions.

Imaging and definition of fusion: CT is increasingly advocated to adjudicate fusion when radiographs are equivocal, with studies correlating percent osseous bridging on CT to clinical stability. Several authors suggest that >50% bridging across the intended fusion plane is a pragmatic threshold. Standardizing CT planes and measurement methods reduces inter-observer variability and should be adopted in future comparative studies.

Adverse events and donor-site morbidity: Reports of BMAC harvest from the iliac crest document low rates of persistent pain or complications; calcaneal harvest appears safe in experienced hands. Fibular harvest requires careful planning to avoid ankle instability; when performed as part of a transfibular approach or with limited segments, functional morbidity is minimal in most series.

## 4. Discussion

The present case extends the concept of a biological intramedullary fibular graft—well established in tibial nonunion—to the domain of ankle arthrodesis revision [20].

Mechanically, the intramedullary fibular strut spans the tibiotalar canal along the longitudinal axis of the construct, functioning as a central load-sharing element that enhances axial stiffness and reduces micromotion at the arthrodesis interface. By occupying the medullary canal, it improves resistance to both shear and rotational forces, particularly in revision settings where bone stock may be compromised. This axial positioning also allows compression forces generated by screw fixation to be transmitted directly through the graft–host interface, promoting mechanical stability across the fusion site [19,22]. Biologically, the autologous fibular graft provides a substantial endosteal and cortical surface area in intimate contact with bleeding host bone, facilitating creeping substitution, revascularization, and progressive graft incorporation. Its structural cortical framework offers osteoconductive guidance, while decortication enhances vascular ingrowth and cellular migration, thereby creating a favorable microenvironment for bone remodeling and consolidation [23]. Therefore, the intramedullary fibular autograft provides both structural mechanical support, acting as a biological nail to enhance axial and rotational stability, and biological stimulus, serving as a scaffold for osteoconduction and progressive incorporation across the fusion site. Augmentation with cancellous ICBG and BMAC adds osteogenic cells and inductive signals to accelerate integration [21].

From the literature, three themes emerge.

First, revision TTC arthrodesis remains at materially higher risk of nonunion than primary fusion, justifying more assertive biological-mechanical strategies [24,25,26]. Nonunion after TTC arthrodesis represents one of the most challenging complications in foot and ankle surgery, with reported rates ranging from 5% to 40% depending on patient comorbidities, technique, and fixation method [27,28]. Revision procedures, in particular, carry significantly higher failure rates due to poor local vascularity, bone loss, and scarring that compromise the biological environment for fusion [29]. The search for a reliable method to enhance osteogenesis while maintaining adequate mechanical stability remains ongoing [30,31,32].

Second, BMAC consistently demonstrates a favorable safety profile and biologic plausibility; although high-level comparative data are limited, its risk-benefit balance is attractive in complex revisions [8,33,34]. In addition to the structural graft, the combination with cancellous iliac crest autograft and bone marrow aspirate concentrate (BMAC) provides an optimal biological environment. BMAC contains mesenchymal stem cells, growth factors, and osteoinductive cytokines that have been shown to enhance fusion rates in foot and ankle arthrodesis [35,36]. Several studies have reported improved radiographic healing and shorter time to union when BMAC is used as an adjunct to conventional grafts.

Third, while fibular onlay struts are widely used, the proportional meta-analysis indicates no routine reduction in nonunion in primary series—suggesting that augments should be individualized rather than applied indiscriminately. Traditional biological strategies for revision ankle fusion include cancellous autograft from the iliac crest, fibular onlay grafts, and, in selected cases, vascularized bone transfers. Most published techniques describe the use of fibular struts placed laterally or posteriorly as onlay supports to increase contact surface and mechanical rigidity. However, these configurations offer limited integration with the medullary canal and depend largely on periosteal incorporation rather than endosteal osteogenesis. Compared with traditional onlay fibular grafts, the intramedullary fibular graft provides superior axial and rotational stability by spanning the fusion site within the tibial and talar canals. Its central placement enhances compression at the arthrodesis interface and promotes a favorable biological environment for osteointegration. Unlike onlay grafts, which mainly provide lateral support and may be less osteogenic, this technique achieves direct endosteal contact, effectively transforming the fibula into a “biological intramedullary nail” that delivers both mechanical support and osteoconductive guidance. To our knowledge, this report represents the first documented use of a decorticated intramedullary fibular autograft in the setting of ankle arthrodesis nonunion, offering a distinct conceptual advantage in revision or complex cases.

From a biomechanical standpoint, the intramedullary graft offers a central axis of load transmission, reducing shear forces at the fusion site compared with traditional onlay grafts. This may also explain the absence of complications or graft collapse in our case. Nevertheless, potential limitations include technical demand, the need for precise canal preparation, and the theoretical risk of compromising the distal tibial or talar blood supply.

Our patient demonstrated progressive consolidation on both radiographs and CT scans within 3 months, consistent with solid fusion, and achieved pain-free weight-bearing (Figure 5). This favorable outcome highlights the synergistic potential of combining mechanical stability (through intramedullary fibular placement) and biological stimulation (through autograft and BMAC).

The review conducted as part of this study reinforces these observations. Most available data on revision TTC arthrodesis emphasize the role of autologous grafting but rarely specify the graft configuration. Reported union rates after revision arthrodesis vary widely—from 60% to 90%—and are often lower when hardware removal, infection, or bone loss are present [32]. The absence of standardized criteria for fusion assessment further complicates the interpretation of results. In this regard, our review underscores the growing consensus that CT-based evaluation provides the most reliable method for detecting trabecular bridging and should be adopted as the gold standard for postoperative assessment [9].

This case highlights the technical feasibility and biological rationale of intramedullary fibular autografting as a revision option for tibiotalar arthrodesis nonunion. The technique leverages fully autologous materials, thereby minimizing immunologic risk and eliminating the need for bone morphogenetic proteins (BMP-2), which are costly and not universally available. Moreover, it offers additional axial support within the fusion canal while promoting osteogenesis through combined structural grafting, cancellous autograft, and BMAC. The successful radiographic consolidation and pain-free functional recovery observed in this patient underscore the potential of this approach in managing complex nonunions with compromised biological environments.

However, this report has several inherent limitations. First, it describes a single-patient experience without a control group, which limits the generalizability of the findings and precludes any comparative conclusions regarding superiority over established techniques. Second, the follow-up period, although sufficient to document radiographic consolidation, does not allow assessment of long-term durability, maintenance of alignment, or late complications such as graft resorption, hardware failure, or adjacent joint degeneration. Third, part of the theoretical rationale for intramedullary fibular use is extrapolated from long-bone nonunion literature, which may not fully replicate the unique biomechanical and biological environment of the hindfoot. Moreover, functional outcomes were not evaluated using standardized patient-reported outcome measures, and advanced quantitative CT metrics were not systematically applied to measure fusion mass or graft incorporation.

Despite these constraints, this case provides proof-of-concept evidence supporting the feasibility and biological plausibility of combining an intramedullary fibular autograft with cancellous iliac crest graft and BMAC in revision TTC nonunion.

Future research should include larger prospective cohorts with standardized surgical protocols and CT-adjudicated fusion assessment. Comparative studies against traditional onlay grafts, structural allografts, or revision intramedullary nailing would help clarify relative biomechanical advantages. Additionally, quantitative evaluation of graft incorporation, time to union, patient-reported functional outcomes, and long-term survivorship analyses are warranted to better define indications, safety profile, and reproducibility of this biological–mechanical strategy.

## 5. Conclusions

The use of an intramedullary fibular autograft combined with cancellous iliac crest autograft and BMAC represents a feasible and biologically sound strategy for managing revision TTC arthrodesis nonunion. By positioning the fibular graft within the tibial and talar canals, this technique provides immediate axial and rotational stability while maintaining intimate host–graft contact, effectively functioning as a “biological intramedullary nail.” The addition of cancellous autograft and BMAC further enhances the osteogenic and osteoconductive environment, promoting fusion without relying on recombinant BMP-2, which may be unavailable or costly in many settings. Importantly, this approach uses entirely autologous materials, reducing potential immunogenic or donor-site complications associated with allografts or synthetic substitutes. While our case demonstrates promising technical feasibility and clinical outcome, larger, CT-adjudicated series are warranted to more rigorously validate the safety, effectiveness, and specific indications of this technique in revision or complex TTC arthrodesis scenarios.

## Figures and Tables

**Table 1:** Summary of key studies on fibular grafts and BMAC relevant to ankle fusion and long-bone nonunion.

Author (Year)	Study Design	Technique/Adjunct	Clinical Setting	Key Outcomes
Bernasconi (2023) [16]	Systematic review/meta-analysis	Fibular onlay graft	Primary TTC arthrodesis	No significant reduction in nonunion vs. screws + cancellous graft
Yadav (2018) [20]	Case series	Intramedullary fibular strut	Long-bone nonunion	Union rates often >85–90%
Glenn (2021) [13]	Narrative review	BMAC	Foot and ankle surgery	Favorable safety profile; promising union in high-risk fusions
Elattar (2024) [15]	Prospective study	Iliac crest BMAC harvest	Foot/ankle fusion	Low donor-site morbidity
Hernigou (2005) [21]	Case series	Percutaneous bone marrow grafting	Long-bone nonunion	Union correlated with progenitor cell dose

## Data Availability

The data presented in this study are available within the article. No new datasets were generated or analyzed beyond those reported in the manuscript.
